# Purinergic Effects on Na,K-ATPase Activity Differ in Rat and Human Skeletal Muscle

**DOI:** 10.1371/journal.pone.0091175

**Published:** 2014-03-10

**Authors:** Carsten Juel, Nikolai B. Nordsborg, Jens Bangsbo

**Affiliations:** 1 Department of Biology, University of Copenhagen, Copenhagen, Denmark; 2 Department of Nutrition, Exercise and Sport, University of Copenhagen, Copenhagen, Denmark; University of California Merced, United States of America

## Abstract

**Background:**

P2Y receptor activation may link the effect of purines to increased maximal *in vitro* activity of the Na,K-ATPase in rat muscle. The hypothesis that a similar mechanism is present in human skeletal muscle was investigated with membranes from rat and human skeletal muscle.

**Results:**

Membranes purified from rat and human muscles were used in the Na,K-ATPase assay. Incubation with ADP, the stable ADP analogue MeS-ADP and UDP increased the Na^+^ dependent Na,K-ATPase activity in rat muscle membranes, whereas similar treatments of human muscle membranes lowered the Na,K-ATPase activity. UTP incubation resulted in unchanged Na,K-ATPase activity in humans, but pre-incubation with the antagonist suramin resulted in inhibition with UTP, suggesting that P2Y receptors are involved. The Na,K-ATPase in membranes from both rat and human could be stimulated by protein kinase A and C activation. Thus, protein kinase A and C activation can increase Na,K-ATPase activity in human muscle but not via P2Y receptor stimulation.

**Conclusion:**

The inhibitory effects of most purines (with the exception of UTP) in human muscle membranes are probably due to mass law inhibition of ATP hydrolysis. This inhibition could be blurred in rat due to receptor mediated activation of the Na,K-ATPase. The different effects could be related to a high density of ADP sensitive P2Y_1_ and P2Y_13_ receptors in rat, whereas the UTP sensitive P2Y_11_ could be more abundant in human. Alternatively, rat could possesses a mechanism for protein-protein interaction between P2Y receptors and the Na,K-ATPase, and this mechanism could be absent in human skeletal muscle (perhaps with the exception of the UTP sensitive P2Y_11_ receptor).

**Perspective:**

Rat muscle is not a reliable model for purinergic effects on Na,K-ATPase in human skeletal muscle.

## Introduction

Purines (ATP and ADP) are released from muscle tissue to the extracellular space during contractions [1] and are therefore potential signalling molecules. Extracellular purines can activate membrane bound G protein-coupled receptors (P2Y receptors) in non-muscle tissue, which causes increased Na,K-ATPase activity [2]. In rat soleus muscle, extracellular purines (0.1 to 1 mM) augments Na,K-ATPase activity, which may be related to stimulation of P2Y_1_ receptors [3]. In support of this the Na,K-ATPase activity in membranes isolated from rat muscle is increased by purinergic stimulation and inhibited by P2Y receptor antagonists [4]. Based on the action of agonists and antagonists it was suggested that the purinergic response in rat muscle membranes involves P2Y_1_, P2Y_3/4_ and P2Y_13_ receptors [4]. The purines increase both the Na^+^ affinity (reduced *K_m_*) and the maximal in vitro capacity (*V_max_*) of the Na,K-ATPase by two independent mechanisms: a P2Y receptor-mediated increase in maximal activity, and a receptor independent, but phosphorylation dependent, increase in Na^+^ affinity [4].

P2Y receptor depend stimulation of Na,K ATPase has been reported for rat muscle [4], it therefore appears likely that a similar mechanism exist in human muscle. However, human skeletal muscle express P2Y_4_ and P2Y_11_ in the plasma membrane [5] whereas P2Y_1,2,4,6,13_ is expressed at the mRNA level in rat muscle [6], [7], [8], [9], [10]. Thus, signalling via P2Y receptor activation may differ between rat and human skeletal muscle.

The pathways from P2Y receptor activation to changes in Na,K-ATPase activity are not known. However, activation of phospholipase C (PLC) appears to be an intermediate step [3], but the downstream pathway from PLC is unclear.

Muscle incubation with purines results in phosphorylation of both the α and phospholemman (PLM) subunits of the Na,K-ATPase in rat [4], which suggests that kinase activation is involved. PLM phosphorylation is primarily caused by PKA and PKC [11]. Additionally, PLM phosphorylation occurs in human muscle as a consequence of contraction and may be related to PKC activation as observed in mouse muscle [12]. However, it is not known if PKC activation causes increased Na,K pump activity in human muscle.

The aim of the present study is to investigate the hypotheses that 1) incubation with activators of purinergic receptors cause increased Na,K-ATPase activity in human skeletal muscle tissue 2) PKA/PKC activation causes increased Na,K-ATPase activity in human skeletal muscle and is therefore a potential mechanism by which P2Y receptor activation increases Na,K-ATPase activity in human muscle.

## Materials and Methods

### Ethical Approval

Six healthy male volunteers (Age 24.9±1.3 year) participated in the study. The human study was approved by the regional ethics committee for the capital region of Denmark (H-A-2009-016). These samples have been used in another study [13]. All subjects gave written informed consent to participate. All animal handling was conducted in accordance with Danish Animal Welfare Regulations. The animal study was approved (P13-073) by Department of Experimental Medicine - International Animal Care and Use System. The animal experiments used male Wistar rats with a body weight of 120–150 g. Rats were provided with unlimited food and water, and were kept under a 12/12-h dark/light cycle.

### Muscle Material

Humans: After resting for 30 min, a muscle biopsy was obtained from m. vastus lateralis under local anaesthesia (Xylocain 20 mg/ml; AstraZeneca) using a modified Bergström needle with suction. The muscle tissue was rapidly frozen (<30 s) in liquid nitrogen and stored at −80°C until further analysis.

Rats: Only white vastus lateralis muscles (fast twitch fibres) were used for the present experiments, but it has previously been demonstrated that purinergic activation of the Na,K-ATPase exists in slow twitch muscles [4].

### Muscle Preparations and Membrane Fractionation

All sample preparations were conducted at 4°C, unless otherwise stated. The sample protein content was determined using a bovine serum albumin (BSA) standard (DC protein assay, Bio-Rad, CA, USA). After mincing, muscle were homogenized for 30 s (Polytron PT 2100) in 250 mM mannitol, 30 mM L-histidine, 5 mM EGTA, and 0.1% deoxycholate, pH 6.8. The sample (crude homogenate) was centrifuged at 3000×*g* for 30 min, and the resulting supernatant was centrifuged at 190,000×*g* spinning for 90 min (at 4°C). The final pellet was re-suspended in assay buffer (see below), and used for activity measurements. The membrane purification above is necessary to remove background activity due to other ATPases.

### Measurement of Na^+^-stimulated Na^+^,K^+^-ATPase Activity

Na^+^-stimulated Na,K-ATPase activity was determined by measuring ATP hydrolysis. Released P_i_ was detected using a malachite based Biomol Green reagent (Biomol # AK-111, Enzo Life Sciences) as previously described [4], [12]. Samples were suspended in assay buffer (10 mM KCl, 5 mM MgCl_2_, 50 mM Tris-base, 5 mM EGTA, pH 7.4). Each sample contained 2–10 µg of protein. The Na^+^ concentration was either 0, 5 or 40 mM (the ionic strength was kept constant by substituting NaCl with choline chloride). 40 mM Na^+^ has previously been shown to fully activate the Na^+^ dependent Na,K-ATPase activity [4], [13]. Graphs demonstrating the Na^+^-dependency of the ATPase activity have been published previously [13]. After 5 min preincubation at 37°C, the reaction was started by adding Mg-ATP to a final concentration of 0.5 mM, and after 30 min, the reaction was terminated by adding 1 mL Biomol Green reagent. After 30 min, absorbance was read at 620 nm and [P_i_] was calculated from a standard curve. All samples were run in triplicate and the ATPase activity at 0 mM Na^+^ was subtracted from the value at 5 or 40 mM Na^+^, consequently only Na^+^ dependent activity was quantified. Previous studies in rat membranes demonstrated that Na^+^-stimulated activity was completely inhibited by preincubation with 2 mM of the specific Na,K-ATPase inhibitor ouabain [14].

### Statistics

The mean Na,K-ATPase activity for each series of experiments were compared using unpaired t-test (SigmaPlot software). The experiments were carried out in pairs; one control experiment was always carried out with an experiment using a test solution (purine or purine+inhibitor). Thus all ATPase experiments had their own control experiment. P<0.05 was considered to be statistically significant.

## Results

**Figure 1:**
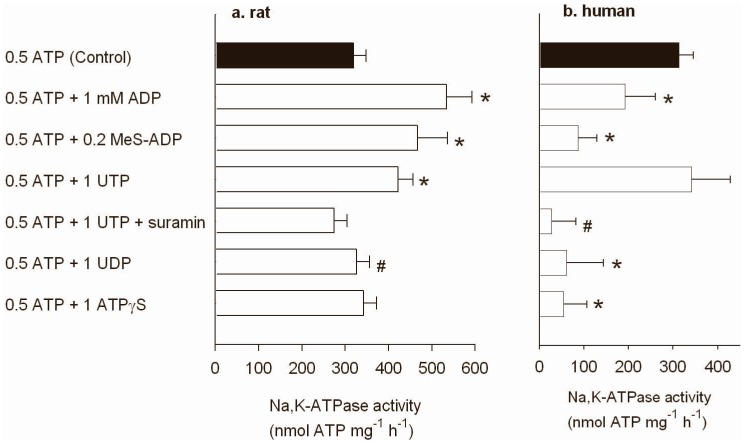
Comparison of the effects of purines on Na^+^ dependent Na,K-ATPase activity in membranes from rat and human skeletal muscle. The assay was performed at 0.5(control, dark column). **a.** Rat muscle. The bars represent the Na^+^ stimulated Na,K-ATPase activity (+SEM) at 40 mM Na^+^ (calculated from the difference between the activity at 0 and 40 mM Na^+^) after pre-incubation with purines (ADP, MeS-ADP, UTP, and UDP) in the presence of 0.5 mM ATP. Mean+SE shown. *indicates different from the activity with 0.5 mM ATP. #indicates different from UTP, n = 8. **b.** Human muscle. The effect of ADP, MeS-ATP, UTP, ATPγS, and UDP) in the presence of 0.5 mM ATP and 40 mM Na^+^ (control). In one series of experiments the P2Y antagonist suramin (100 µM) was added together with UTP, n = 6.

The data below represents Na,K-ATPase activity at 40 mM Na^+^. In rat muscle ADP (1 mM), MeS-ADP (0.2 mM), and UTP (1 mM) increase (P<0.05) Na,K-ATPase activity with ∼67, 46, and 32%, respectively, compared to the activity with 0.5 mM ATP (control). Incubation with UDP (1 mM) and ATPγS (1 mM) had no effect (Fig. 1a). The stimulatory effect of ADP in rat was completely inhibited by pre-incubation with the P2Y receptor antagonist suramin (100 µM) (data not shown). Likewise, the stimulatory effect of UTP was inhibited with suramin.

Similar experiments performed with membranes from human skeletal muscle resulted in different results: preincubation with ADP, MeS-ADP and UDP decreased the Na,K-ATPase activity compared to the activity with 0.5 mM ATP (Fig. 1b). Since P2Y11 receptors have been reported to be exclusively present in humans, additional experiments were performed with the P2Y11 receptor agonist ATPγS. Incubation with ATPγS (1 mM) decreased the Na,K-ATPase activity in membranes from human muscle compared to control.

The Na,K-ATPase activity was not inhibited with UTP (1 mM), however a reduced Na,K-ATPase activity was obtained if the inhibitor suramin (100 µM) was applied together with UTP (Fig. 1B).

**Figure 2:**
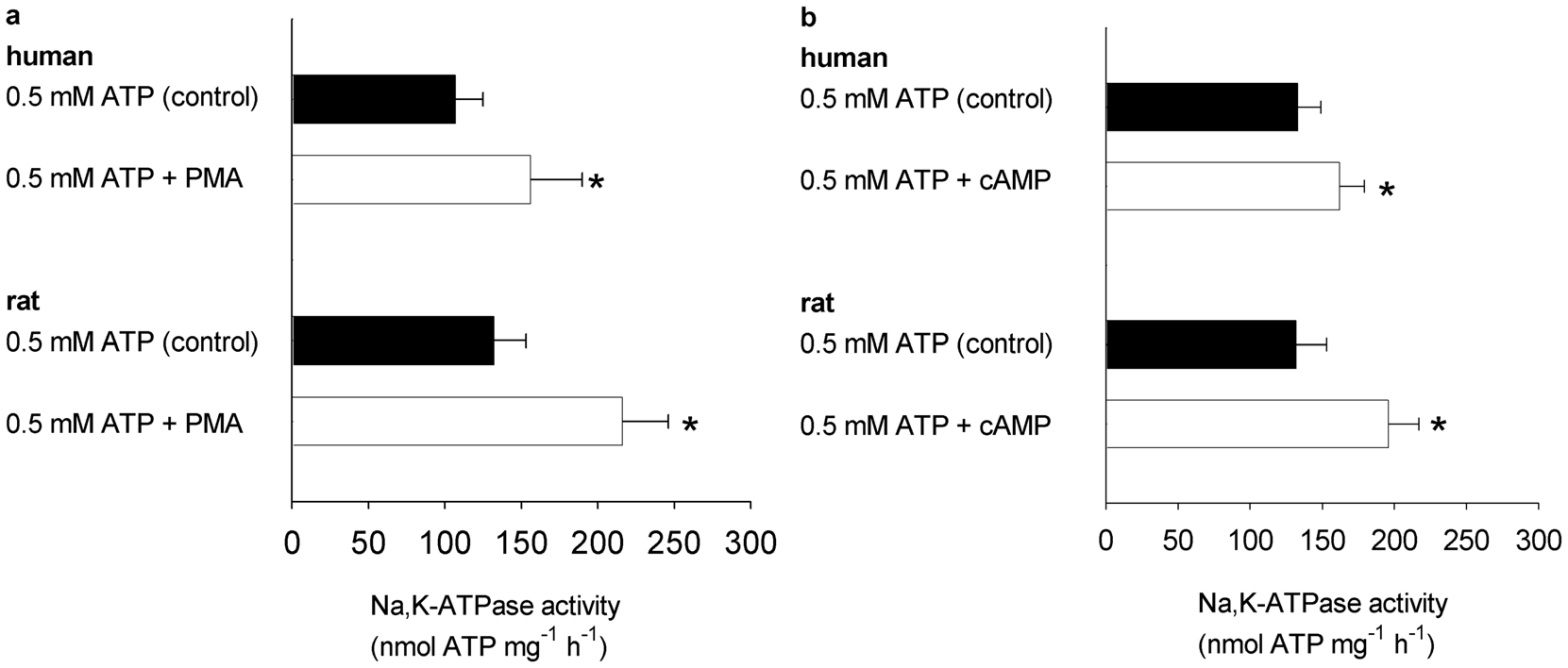
Effect of PKA and PKC stimulation on Na,K-ATPase activity in membranes from rat and human skeletal muscle. a. Effect of the PKC activator PMA (phorbol 12-myristate 13-acetate) in membranes from rat and human skeletal muscle tested at 5 mM Na^+^, n = 6, SEM shown. **b.** Effect of the PKA activator cAMP (1 mM) in membranes from rat and human skeletal muscle tested at 5 mM Na^+^, n = 6 *: significantly different from control.

The following experiments were performed to compare the effects of PKA and PKC activation on the Na,K-ATPase activity in membranes from rat and human skeletal muscle. The experiments were performed at a low Na^+^ concentration (5 mM) to allow detection of the combined effect of changes in Na affinity (*K_m_*) and *V_max_*. Therefore, the setup does not discriminate between changes in *K_m_* and *V_max_*. The PKA activator cAMP (1 mM) significantly increased the Na,K-ATPase activity at 5 mM Na^+^ both in rat (+64%) and human (+46%) samples (Fig. 2a).

Similarly, the protein kinase C activator PMA (phorbol 12-myristate 13-acetate) (100 nM) significantly increased Na,K-ATPase activity at 5 mM Na^+^ both in membranes from rat (+48%) and human (+22%) skeletal muscle (Fig. 2b).

## Discussion

The major finding in the present study was that incubations with purinergic receptor activators caused inhibition of Na,K-ATPase activity in membranes purified from human skeletal muscle. In contrast, Na,K-ATPase activity was increased by purinergic stimulation in glycolytic rat muscle. Additionally, PKA activation by cAMP incubation and PKC activation by PMA both increased Na, K-ATPase activity in human and rat skeletal muscle.

### Effect of Purines on Na,K-ATPase Activity

The Na,K-ATPase assay used in the present study is based on membrane purification to reduce the background activity of other ATPases. The different purine sensitivity could therefore be the result of different purifications of Na,K-ATPase proteins from rat and human skeletal muscle. However, the total numbers of Na,K-ATPase proteins (quantified with ^3^H-ouabain binding) in rat and human skeletal muscle are of the same magnitude [15]. The ATPase activity in membranes purified from rat and human muscle is also similar (Figure 1 and 2), which indicates that the efficiency of membrane purification in rat and human skeletal muscle is similar. In addition, we have previously demonstrated similar recovery (∼5–6% of the isoform content in the muscle homogenate) of the α1 and α2 isoform [4]. It is therefore unlikely that the species variation obtained in the present study is due to differences in membrane purification.

In contrast to the study hypothesis, ADP, MeS-ADP, and ATPγS incubation resulted in Na,K-ATPase inhibition in membranes purified from human skeletal muscle. However, ADP, MeS-ADP and UTP incubations induced an increase of rat muscle Na,K-ATPase activity, which confirms previous findings [4]. In addition, ATPγS had no effect in rat muscle. From these findings it is clear that the purinergic effect on Na,K-ATPase activity is species dependent. It should be noted that all incubations contained 0.5 mM ATP which per se increases the ATPase activity ∼30% [4] and as such represent a physiological situation.

One explanation for the different effects of purinergic incubations between species may be related to a difference in P2Y receptor isoform expression between rat and human muscle. The P2Y_1_ and P2Y_13_ receptors are expressed in rat muscle and are highly sensitive to ADP [6], [8], [9]. It is less clear which isoforms that are expressed in human muscle. Moderate amounts of P2Y_4_ in the sarcolemma and a low density of intracellular P2Y_11_ have been reported [5], but the human P2Y_11_ receptor appears to be less ADP sensitive [6]. The present incubation with the P2Y_11_ agonist ATPγS caused an inhibition of Na,K-ATPase activity in human muscle, suggestion that P2Y_11_ receptor stimulation is not important for regulation of Na,K-ATPase activity during contractions. Moreover, incubation with UTP did not affect Na,K-ATPase activity, but simultaneous incubation with UTP and the P2Y antagonist suramin reduced Na,K-ATPase activity, suggesting that P2Y receptors are involved in the response to UTP. The lacking effect of UTP can be seen as a balance between a direct inhibition (see below) mediated by UTP and a receptor mediated stimulation.

Only few studies have discriminated between P2Y receptor expression in muscular and microvascular compartments. This may be of importance, as illustrated by the finding of high levels of P2Y_2_ mRNA in muscle samples where immunohistochemistry analyses revealed that the receptor is primarily expressed in the microvascular compartment [16]. PCR measurements must therefore be interpreted with caution.

Based on mass law action it can be suggested that an increased purine concentration will inhibit the hydrolysis of ATP. However, previous studies in rat have demonstrated that purines increase Na,K-ATPase *V_max_* via P2Y receptors [4]. Thus, the effect of purines can be described as a balance between inhibition and stimulation.

Alternatively, the difference in the response to purinergic stimulation in rat and human skeletal muscle could be due to differences in the apparent protein-protein interaction between membrane bound P2Y receptors, membrane bound intermediates and the Na,K-ATPase, which has been suggested for rat muscle [4].

### Effect of PKA and PKC Activation on Na,K ATPase Activity

This study also demonstrated that both PKA and PKC activation can stimulate the Na,K-ATPase in rat and human skeletal muscle. The different responses to purines could therefore not be related to differences in sensitivity and abundance of these kinases.

However, it must be noted that there is no proof of a direct coupling between P2Y receptors and PKA/PKC activation, other kinases may be involved in the purinergic regulation of Na,K-ATPase activity. The different response to purines in rat and human could therefore also be related to differences in other signal pathways.

In conclusion, the present results demonstrate that Na,K-ATPase activity is inhibited by purinergic incubation, which is in contrast to the purinergic stimulation of Na,K-ATPase activity in rat skeletal muscle. These data demonstrate that conclusions based on experiments with one model system, can not be uncritically applied to another system.
